# A comprehensive review of AAV-mediated strategies targeting microglia for therapeutic intervention of neurodegenerative diseases

**DOI:** 10.1186/s12974-024-03232-2

**Published:** 2024-09-19

**Authors:** Livia Zhou, Yafeng Wang, Yiran Xu, Yaodong Zhang, Changlian Zhu

**Affiliations:** 1 Henan Neurodevelopment Engineering Research Center for Children, Children’s Hospital Affiliated to Zhengzhou University, Henan Children’s Hospital Zhengzhou Children’s Hospital, Zhengzhou, 450018 China; 2 Henan Key Laboratory of Child Brain Injury and Henan Pediatric Clinical Research Center, Institute of Neuroscience and The Third Affiliated Hospital of Zhengzhou University, Zhengzhou, Henan Province China; 3 Center for Brain Repair and Rehabilitation, Institute of Neuroscience and Physiology, University of Gothenburg, Gothenburg, Sweden

**Keywords:** Microglia, Adeno-associated virus (AAV), Neurodegenerative diseases, Gene Therapy, Microglia Tropism

## Abstract

Neurodegenerative diseases pose a significant health burden globally, with limited treatment options available. Among the various cell types involved in the pathogenesis of these disorders, microglia, the resident immune cells of the central nervous system, play a pivotal role. Dysregulated microglial activation contributes to neuroinflammation and neuronal damage, making them an attractive target for therapeutic intervention. Adeno-associated virus (AAV) vectors have emerged as powerful tools for delivering therapeutic genes to specific cell types in the central nervous system with remarkable precision and safety. In the current review, we discuss the strategies employed to achieve selective transduction of microglia, including the use of cell-specific promoters, engineered capsids, and microRNA (miRNA) strategies. Additionally, we address the challenges and future directions in the development of AAV-based therapies targeting microglia. Overall, AAV-mediated targeting of microglia holds promise as a novel therapeutic approach for neurodegenerative diseases, offering the potential to modify disease progression and improve patient outcomes.

## Introduction

Neurodegenerative diseases represent a class of debilitating disorders characterized by progressive degeneration and dysfunction of the central nervous system, leading to cognitive decline, motor impairment, and ultimately, profound disability. Neurodegenerative diseases include both common diseases and rare diseases, such as Alzheimer’s disease (AD) and Huntington’s disease (HD), respectively, collectively affecting millions worldwide. With aging populations on the rise globally, the burden of neurodegenerative diseases is escalating, posing significant challenges to healthcare systems and economies worldwide [1]. Current therapeutic strategies often offer limited symptomatic relief but fail to halt or reverse disease progression. Thus, there is an urgent need for innovative therapeutic approaches targeting underlying disease mechanisms, ranging from neuroprotective agents to gene therapies and regenerative medicine, to address the unmet medical needs of patients and alleviate the societal burden of these devastating disorders [2, 3].

### Neurodegenerative diseases associated with microglia dysfunction

Microglia are crucial immune cells in the central nervous system (CNS), and their dysfunction contributes to the pathogenesis of various neurodegenerative diseases [4]. Some diseases are specifically caused by gene mutations occurring solely within microglia, such as colony-stimulating factor 1 receptor (*CSF-1R*) mutation-induced neurodegenerative diseases [5]. CSF-1R is a receptor protein found on the surface of microglia in the CNS, that binds to colony-stimulating factor 1 (CSF-1) and interleukin-34 (IL-34) [6]. Activation of CSF-1R is crucial for the development, survival, and function of microglia in the CNS [6, 7].

Hereditary Diffuse Leukoencephalopathy with Spheroids (HDLS) is a rare inherited neurodegenerative disorder characterized by a progressive decline in cognitive function, movement abnormalities, and changes in white matter in the brain, particularly in the frontal and temporal lobe [8]. Mutations in the *CSF-1R* gene have been identified as a genetic cause of HDLS. In individuals with HDLS caused by *CSF-1R* mutations, the normal functioning of microglia is disrupted, resulting in chronic inflammation, impaired clearance of cellular debris, and damage to white matter in the brain [5, 9]. Currently, there is no cure for HDLS, and treatment focuses on managing symptoms and supportive care.

Most microglia-related disorders typically involve complex interactions between multiple cell types and genetic factors rather than being solely attributed to microglial gene mutations. Due to the intricate pathology of these neurodegenerative diseases, there is currently no effective cure, and treatments primarily focus on managing symptoms. Recently, new treatments have emerged; for instance, Aβ antibody therapies for AD were approved by U.S. Food and Drug Administration (FDA) [10]. Additionally, cell and gene therapies are under development in clinical trials for other diseases such as Parkinson’s disease (PD), HD, and Amyotrophic Lateral Sclerosis (ALS) [11–13]. However, the efficacy of these therapies remains limited, potentially due to the complex progression stages of these diseases and the timing of diagnosis.

Mutations in genes expressed in microglia or affecting their function can contribute to the pathogenesis of certain neurological disorders. For instance, mutations in the *TREM2* gene, predominantly expressed on microglia, are associated with several neurodegenerative disorders, including Nasu-Hakola disease, frontotemporal dementia (FTD), and AD [14, 15]. Mutations in the *GRN* gene are a significant cause of FTD, where *GRN* mutations in microglia result in increased microglial activation, neuroinflammation, and impaired lysosomal function, thereby contributing to FTD pathology [16]. Additionally, mutations in the *LRRK2 *gene represent the most common genetic cause of familial and sporadic PD [17]. Studies have shown that *LRRK2* is highly expressed in microglia and is involved in microglial inflammatory responses; mutations in *LRRK2* can lead to abnormal inflammatory responses in PD [18]. Similarly, the mutant huntingtin protein (mHTT) influences microglial activation and inflammatory responses, with microglia expressing mHTT exhibiting altered cytokine release, contributing to HD [19]. Beyond these examples, genes such as *C9orf72*, *OPTN*, *TBK1*, *SOD1*, *MCP-1*, *CSF1R*, *P2RY12*, *SALL1*, and *CX3CR1* are also potential targets for gene therapy aimed at modulating microglial function.

Understanding the multifaceted roles of microglia and their genetic underpinnings is crucial for unraveling the complexities of these disorders and developing targeted therapeutic strategies. In AD, studies have revealed that most risk genes are highly expressed in microglia, indicating their critical role in disease progression [20]. Dysfunctional microglia contribute to AD by becoming less effective at clearing amyloid-beta plaques, releasing pro-inflammatory cytokines and neurotoxic substances, promoting tau pathology, and impairing neuronal activity [21–23]. In PD, microglia become overactivated, releasing pro-inflammatory cytokines and neurotoxic substances that exacerbate the loss of dopaminergic neurons in the substantia nigra [24, 25]. This chronic inflammation further disrupts the regulatory mechanisms of microglial activity, impairing their ability to clear alpha-synuclein aggregates, a key pathological feature of PD [26, 27]. In FTD and ALS, dysfunctional microglia contribute to the inflammatory response and reduce the clearance of TDP-43, leading to damage in the frontal and temporal lobes and motor neurons in the brain and spinal cord, respectively [28, 29]. In HD, active microglia in the striatum contribute to inflammation and the loss of neuronal support [30, 31]. Furthermore, microglia expressing mHTT become more aggressive towards neurons than wild-type microglia upon activation [19]. Notably, the contribution of dysfunctional or activated microglia varies across different diseases, suggesting that microglia-targeted therapies may need to be disease-specific.

These examples illustrate instances where mutations or dysfunctions in microglia are involved in the broader disease process. However, it’s crucial to note that research into microglial genetics and their role in disease pathogenesis is ongoing, and future discoveries may shed more light on diseases directly caused by gene mutations within microglia or microglia dysfunction. Therefore, gene therapies targeting microglia may provide a promising therapeutic strategy for these challenging-to-treat diseases. Moreover, advancing techniques such as gene editing and gene delivery systems may offer novel approaches to correct aberrant microglial function and restore CNS homeostasis in various neurodegenerative disorders.

### AAV therapy for neurodegenerative diseases

**Fig. 1:**
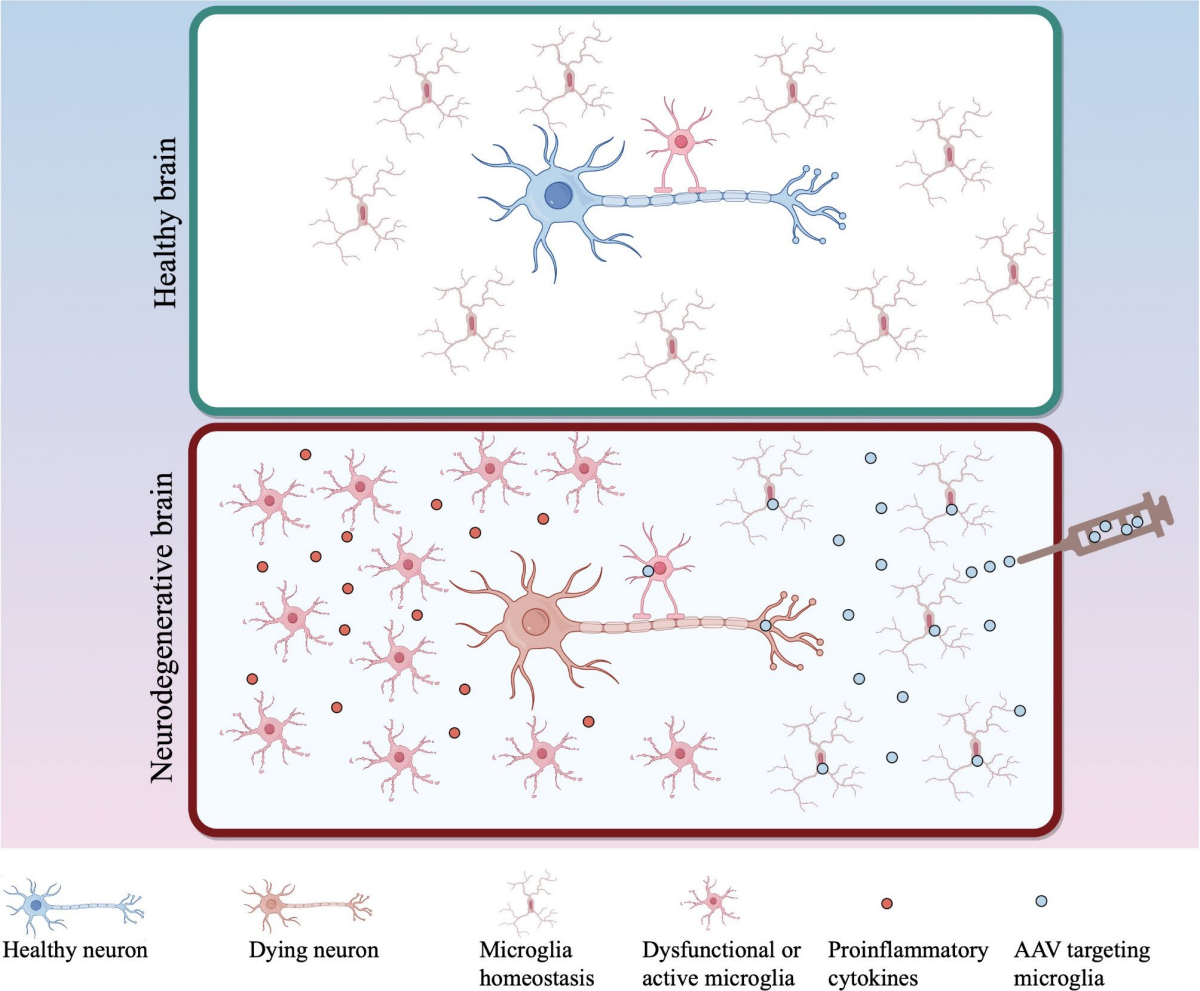
Microglia-targeting AAV therapies for neurodegenerative diseases. An ideal therapeutic recombinant adeno-associated virus (rAAV) with microglia-specific promoters can efficiently and specifically target microglia. This approach aims to treat dysfunctional or overly active proinflammatory microglia, ultimately refining the microenvironment and promoting neuronal survival

AAV vectors have emerged as promising tools for gene therapy in the CNS, with several products reaching clinical approval. Notable examples include the AAV2-based medication Upstaza^®^ (INN: Eladocagene exuparvovec) for aromatic L-amino acid decarboxylase (AADC) deficiency, sanctioned by the European Medicines Agency (EMA) in 2022, and Luxturna^®^ (INN: voretigene neparvovec) for *RPE65* mutation-associated retinal dystrophy, approved by FDA in 2017. Despite these advancements, the majority of current AAV serotypes or variants exhibit tropism primarily towards neurons or astrocytes [32]. As such, there is ongoing research aimed at developing AAV variants with enhanced tropism for microglia [33]. Various strategies are being explored to achieve this objective, with the ultimate goal of improving the specificity and efficacy of gene therapies targeting microglial function in neurological disorders (Fig. 1).

**Fig. 2:**
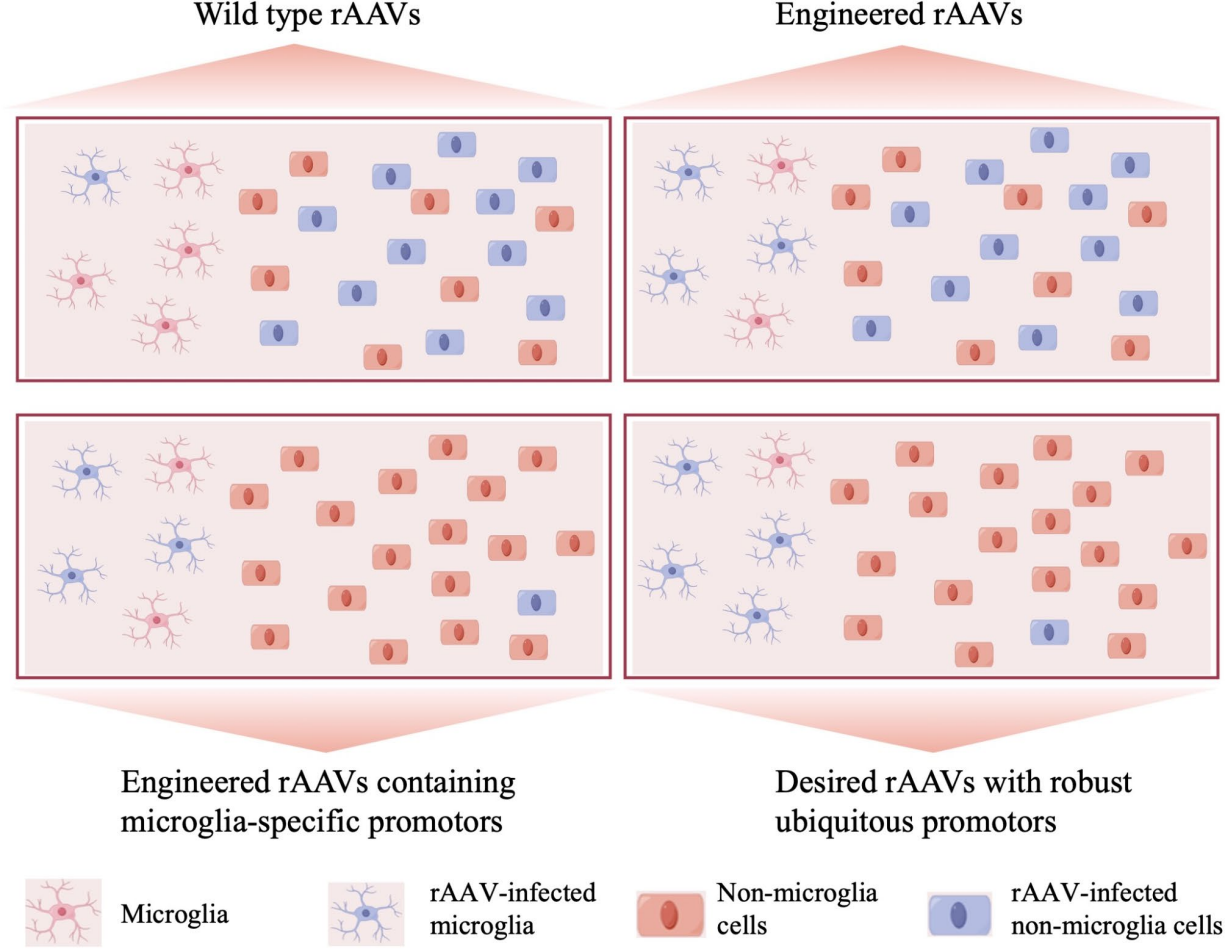
The comparative efficiency of various rAAVs targeting microglia within the brain. In the top-left panel, wild-type rAAV predominantly targets non-microglial cells with limited microglial transduction; the top-right panel depicts an engineered AAV with enhanced tropism towards both microglia and non-microglial cells; the bottom-left panel showcases an engineered rAAV incorporating microglia-specific promoters, while simultaneously incorporating miRNA binding sites specific to non-microglial cells, resulting in targeted microglial transduction; the bottom-right panel demonstrates the efficacy of a desired rAAV variant exhibiting robust tropism towards microglia through the utilization of ubiquitous promoters

One approach involves screening and selecting naturally occurring AAV serotypes or variants that exhibit preferential transduction of microglia. Another strategy involves directed evolution and rational design to engineer AAV capsids with improved affinity for microglial cell surface receptors [34]. For example, engineering AAV capsids to target receptors such as CD11b or CX3CR1, which are highly expressed on microglia, may enhance their tropism for these cells. Furthermore, the development of hybrid capsids, chimeric capsids, and peptide display technologies allows for the generation of novel AAV variants with customized tropism profiles [32, 35]. These engineered AAV vectors can be designed to selectively transduce microglia while minimizing off-target effects on other cell types in the CNS (Fig. 2). The transduction efficiency, defined in this study as the percentage of total cells expressing either indicator genes or the transgene of interest for various AAV serotypes with different promoters is presented in Table 1.

**Table 1:** Efficiency of various AAV serotypes and promoters for targeting microglia in vitro and in vivo

AAV capsids	Dose (vg)	Promoters	Transduction rate		Reference
			In vitro	In vivo (brain)	
AAV2	1E8/chamber	*CBA*	Poor	x	Rosario, et al., 2016
AAV2	1.75E3/cell	*CMV*	≥ 80%	x	Su, et al., 2016
AAV2	unkown	*CMV*	High	x	Cucchiarini, et al., 2003
AAV2	unkown	*F4/80*, *CD68*, *or CD11b*	low	x	Cucchiarini, et al., 2003
AAV2	2.5E10 or 9E10	*CMV*	x	Low (Intracranial)	Bartlett, et al., 1998
AAV2	2E10	*CBA*	x	Low (ICV)	Chakrabarty, et al., 2013
AAV5	1E4/cell	*CMV*	12%	x	Lin, et al., 2022
AAV5	1.75E3/cell	*CMV*	Same level of mRNA expression as AAV2	x	Su, et al., 2016
AAV5	2E10	*CBA*	x	low (ICV)	Chakrabarty, et al., 2013
AAV9	5E5 and 1E6 /cell	*CBA*	low	x	Gong, et al., 2015
AAV9	1E11	*CBA*	x	3% (ICV, in spinal cord)	Gong, et al., 2015
AAV9	1-3E12	*CBA*	x	18% (IV, in spinal cord)	Gong, et al., 2015
AAV9	1.2E13	*CD68*	x	Unkown (Intrathecal, in spinal cord)	Grace, et al., 2016
AAV9	1.95E9-3.9E10	*PKG* or *Iba-1*	x	Unkown (Intracranial)	Okada, et a., 2022
AAV9	1E4/cell	*CMV*	10%	x	Lin, et al., 2022
AAV6	1.75E3/cell	*CMV*	80-fold of mRNA expression as AAV2	x	Su, et al., 2016
AAV8	1.75E3/cell	*CMV*	25-fold of mRNA expression as AAV2	x	Su, et al., 2016
AAV8	1E4/cell	*CMV*	34%	x	Lin, et al., 2022
AAV6TM	1E8/chamber	*CBA*	High mRNA and protein experssion compared with wt AAV6	x	Rosario, et al., 2016
AAV6TM	1E8/chamber	*F4/80* or *CD68*	95%	x	Rosario, et al., 2016
AAV6TM	2E8	*CBA*, *F4/80*, or *CD68*	x	Low (ICV or Intracranial)	Rosario, et al., 2016
AAV6TM	1E8/well	*CD68*	modest	x	Maes, et al., 2021
AAV6TM	1.37E8-1.06E9	*CD68*	x	Low (Subretinal or intravitreal, in eyes)	Maes, et al., 2021
AAV6TM	1E4/cell	*CMV*	3%	x	Lin, et al., 2022
AAV-cMG	1E4/cell	*CMV*	86%	x	Lin, et al., 2022
AAV-cMG	4E10 or 2.5E10	*CMV*	x	80% (Intracranial)	Lin, et al., 2022
AAV9- ALAVPFR, ALAVPFK, HGTAASH, and YAFGGEG	1E12	*CD11b*	x	46.7%, 66.9%, 72.8%, and 80.8%, repectively. (IV)	Young, et al., 2023

### Targeting microglia with wildtype AAV serotypes

Recombinant AAV2 (rAAV2) containing a cytomegalovirus (*CMV*) enhancer/ chicken beta actin (*CBA*) promoter exhibited poor transduction efficiency in primary mouse microglia [36]. Conversely, an alternative study reported a notable 80% transduction efficiency of rAAV2-*GFP* with a *CMV* promotor in primary mouse microglia [37]. Additionally, primary microglia derived from rats exhibited high transduction rates with rAAV2 containing a *CMV* promoter, although switching the promoter to microglial-specific promoters such as *f4/80*, *CD68*, and* CD11b* resulted in a significant decrease in transduction efficiency [38]. Intracranial injection of 9E10 vector genome (vg) wild type AAV2 labeled with Cy3 dye and 2.5E10 vg rAAV2 with a *CMV* promotor demonstrated robust transduction in neurons but negligible transduction in microglia within the hippocampus and the inferior colliculus of the rat brain [39]. Similarly, another investigation observed the absence of microglial transduction following intracerebroventricular (ICV) injection of 2E10vg rAAV2-*GFP* containing *CMV* enhancer/*CBA* promoter in neonatal mice [40]. Overall, the ubiquitous promoter exhibited stronger transduction efficiency in microglia compared to the microglia-specific promoter in rAAV2. The transduction of rAAV2 in microglia appears limited, particularly in vivo.

rAAV5 demonstrated a notable transduction efficiency in primary microglial cultures derived from rat brains; however, when employing microglia-specific promoters, such as *f4/80*, *CD68*, and *CD11b*, the transduction rates were markedly reduced, with rAAV5-*f4/80*-*Laz* achieving a 25% transduction rate, rAAV5-*CD68-Laz* reaching 10%, and rAAV5-*CD11b-Laz* showing minimal transduction, with one cell or less observed in a single field [38]. Notably, the *f4/80* promoter exhibited the strongest expression and specificity in microglia, as evidenced by the robust expression and microglial restriction of rAAV5 when utilizing the *f4/80 *promoter following intracranial injection into the striatum of rat brains [38]. Nevertheless, the extent of microglial transduction remained limited in this study, underscoring the ongoing challenge of achieving widespread microglial transduction. Additionally, other investigations revealed that rAAV5 with a *CMV* promoter successfully transduced primary cultured mouse microglia [33, 37]. Conversely, another study revealed no microglial transduction with ICV injection of rAAV5 containing the *CMV* enhancer and *CBA* promoter in neonatal mice [40]. Overall, among the microglia-specific promoters, the *F4/80* promoter demonstrated the highest transduction efficiency in microglia with rAAV5. However, the overall transduction efficiency remains limited, particularly in vivo.

In a glial cell culture system derived from *Abcd1*^*−/−*^ mice, a model for X-linked adrenoleukodystrophy (X-ALD), 2% of rAAV9-*ABCD1* carrying a *CMV* enhancer/*CBA* promoter successfully transduced microglia [41]. Subsequent ICV and intravenous (IV) injections of 1E11 vg and 1-3E12 vg of rAAV9, respectively, led to microglial transduction in the brain and spinal cord of *Abcd1*^*−/−*^ mice, albeit with limited efficiency, as evidenced by the transduction rates of 3% and 18% of microglia in the spinal cord following ICV and IV administration, respectively [41]. Moreover, another investigation demonstrated that intrathecal delivery of AAV9 with a *CD68* promoter specifically targeted microglia in the spinal cord [42]. However, the findings of this study have been subject to debate due to concerns regarding the quality of immunohistochemical staining techniques [34]. Thus, while rAAV9 demonstrates the potential to transduce microglia in vivo depending on delivery routes, its efficiency remains constrained.

Further enhancements in transduction efficiency and reduction of neuronal and astrocytic transduction were achieved by incorporating *miR-9.T* and *miR-129-2-3p.T* in both physiological and pathological mouse models, including those of LPS-induced neuroinflammation and neurodegenerative diseases [43]. Notably, these microRNAs (miRNAs) are exclusively expressed in non-microglial cells [44–46]. AAV9-*PGK*.*miR-9.T* demonstrated that less than 10% of the total targeting cells were microglia following intracranial injection in the cortex and striatum, with a slightly higher transduction rate observed in the cerebellum at 37%. Injection volumes of 0.5, 1, and 10 µl containing 3.9e12 vg were utilized for the cortex, striatum, and cerebellum, respectively [43], indicating that the *PGK* promoter with *miR-9.T* is not an effective strategy for widespread and specific microglial targeting in the brain. Conversely, employing the same dose of AAV9-*Iba1*.*miR-9.T* resulted in microglia transduction rates of 69%, 86%, and 2% out of the total transduced cells in the striatum, cerebellum, and cortex, respectively. These findings suggest that Iba-1 may serve as a superior promoter combined with miRNA strategy for microglial targeting in certain brain regions such as the striatum and cerebellum [43]. Even though the targeting microglia specificity increased using these strategies, the total number of transduced microglia was still limited.

Various AAV serotypes have been assessed and compared for their ability to transduce primary microglia. For instance, a study demonstrated that rAAV6-*CMV-GFP* and rAAV8-*CMV-GFP* yielded an 80-fold and 25-fold increase in transduction efficiency, respectively, compared to rAAV2-*CMV-GFP* [37]. Additionally, rAAV8-*CMV-mScarlet* and rAAV9-*CMV-mScarlet* achieved transduction rates of 34% and 10%, respectively, in primary cultured mouse microglia [33]. In contrast, investigations reported no transduction of microglia by rAAV serotypes 1 through 10 and rh.10 when utilizing a *CBA* promoter in primary cultured mouse microglia [36]. Furthermore, another study revealed the inefficacy of rAAV1, rAAV7, rAAV8, and rAAV9, with *CMV* enhancer and *CBA* promoter, respectively, in transducing microglia following ICV injection at a dose of 2E10 vg in neonatal mice [40]. Collectively, these findings underscore the limited transduction efficiency of wild-type AAV, particularly evident in vivo experiments, and highlight the high specificity but low transduction efficiency of microglia-specific promoters.

### Engineered AAVs targeting microglia

The Y731F/Y705F/T492V triple-mutant (TM) AAV6 capsid (rAAV6TM), engineered with mutations affecting various surface-exposed serine and tyrosine residues, initially demonstrated efficacy in transducing primary microglia cultures and mixed neuroglial cultures from mouse [36, 47]. However, another study revealed that the transduction efficiency of rAAV6TM was limited to 3% in primary mouse microglia cultures [33]. Subsequent studies revealed microglial transduction by rAAV6TM following ICV injection in neuronal mice and intraparenchymal injection in adult mice, although the majority of transduced cells remained non-microglial [36]. To enhance microglial specificity, the *F4/80* or *CD68* promoter was employed, yet overall transduction efficiency for microglia remained modest [36]. Further efforts with rAAV6TM-*GFP* utilizing a *CD68* promoter for subretinal and intravitreal targeting of retinal microglia yielded a mere 1.5% GFP + cells, with less than 10% of these being microglia, underscoring the challenges in achieving efficient and specific microglial targeting [47]. Despite additional mutations aimed at reducing extracellular matrix binding in rAAV6TM, the overall transduction rate of microglia remained low [47].

Two novel variants engineered from AAV9, designated as AAV-cMG.QRP and AAV-cMG.WPP, through the insertion of a seven-amino-acid sequence into the AAV9 VP1 protein, exhibited high transduction efficiencies of 55% and 75%, respectively, in primary mouse microglia. However, their in vivo transduction rates were limited following intra-striatal and intra-midbrain injections in the mouse brain, utilizing doses of 4E10 and 2.5E10 vg, respectively. Further refinement of AAV-cMG.QRP led to the development of an AAV-cMG variant with an enhanced primary microglia transduction rate of 86%, coupled with the absence of inflammation pathway activation. Notably, intra-striatal and midbrain injections of this optimized variant resulted in the labeling of 80% of microglia with 4E10 and 2.5E10 vg doses, respectively [33]. However, the AAV-cMGs were not selectively transduced microglia but also strongly transduced both neurons and astrocytes. Furthermore, the study utilized Cre-dependent gene expression in AAV and *Cx3cr1*^*CreER*^ transgenic mice instead of a microglial-specific promoter to specifically target microglia, offering a potent tool for manipulating microglia in the brain [33]. Nevertheless, the potential of AAV-cMGs with a microglia-specific promoter in the realm of drug development warrants further investigation.

A novel family of AAV variants, termed the AAV-innate family, has been identified for their high transduction efficiency of microglia following IV administration at a total dose of 1E12 vg per mouse [48]. This AAV-innate family comprises four distinct variants based on AAV9, namely ALAVPFR, ALAVPFK, HGTAASH, and YAFGGEG, demonstrating transduction rates of 46.7%, 66.9%, 72.8%, and 80.8% of all microglia, respectively. Utilizing one of these variants, researchers successfully delivered diphtheria toxin A under the control of the *CD11b* promoter, achieving microglial depletion in the brain following IV injection [48]. Furthermore, the delivery of a short hairpin RNA (shRNA) gene under the control of the *CD11b* promoter led to a notable 50% suppression of gene expression specifically in microglia [48]. While these findings highlight the promising potential of AAV-innate variants for precise modulation of microglial function and gene expression in neurological disorders, further investigation is warranted to validate their efficacy and safety in diverse animal models, including rodents and non-human primates (NHPs).

Overall, the creation of AAV variants with enhanced tropism for microglia holds great promise for advancing gene therapy approaches targeting microglial function in neurological disorders. Continued research in this field is expected to lead to the development of more efficient and specific AAV vectors for therapeutic applications in microglia-related diseases.

### Challenges and future directions

The engineering of AAV capsids for enhanced microglia targeting presents several challenges and prompts future research directions in gene therapy. First, achieving selective transduction of microglia while minimizing off-target effects remains a formidable hurdle. Addressing this challenge necessitates a deeper understanding of the molecular mechanisms underlying microglial interaction with AAV capsids in both resting and activated states. Accordingly, optimizing capsid modifications to enhance microglial transduction efficiency and specificity, such as through rational design or directed evolution approaches, is imperative.

Another strategy to increase microglial specificity is to use microglial-specific promoters, such as *iba-1*, *CD11-b*, *CD68*, *Cx3cr1*, and *F4/80*. However, these promotors are usually much weaker compared to ubiquitous promoters, such as* CBA* and *CMV*, leading to lower levels of transgene expression [49, 50]. Moreover, ensuring absolute specificity is difficult, and microglia-specific promoters may exhibit some off-target expression in other cell types, such as macrophages, due to their shared lineage and similar marker expression [51]. Additionally, AAV vectors have a limited packaging capacity (around 4.7 kb), and incorporating microglia-specific promoters can limit the space available for the therapeutic gene. Furthermore, microglia exhibit heterogeneity across different brain regions and disease states, which can influence promoter activity and complicate the uniform expression of therapeutic genes [52]. miRNAs can enhance the specificity of microglial targeting, as described above. However, they do not increase the overall number of microglia infected by AAV. Additionally, incorporating miRNA binding sites occupies space within the AAV vector, potentially limiting the size of the transgene that can be delivered. Moreover, there is a risk that miRNA binding sites may elicit an unintended immune response.

Elucidating the immune responses elicited by AAV capsids in microglia is critical for ensuring long-term therapeutic efficacy and safety. Some wild-type serotypes have broad immune recognition, and this immune response may cross-react with other AAV variants. Therefore, immunosuppressive agents are often used in clinical practice. Furthermore, it is noteworthy that different delivery routes and different preclinical species may impact microglial targeting, underscoring the importance of refining delivery routes and preclinical models to precisely evaluate the biodistribution, cellular tropism, and therapeutic outcomes of novel AAV capsids targeting microglia. Lastly, none of the microglial-targeting AAVs have undergone clinical trials to date, necessitating further confirmation of safety and efficacy in NHP prior to clinical translation. Overall, advancing the engineering of AAV capsids tailored for microglia targeting represents a pivotal avenue for the development of effective gene therapies for neurodegenerative diseases and neurological disorders.

## Summary

The escalating global aging demographic underscores the pressing medical necessity posed by neurodegenerative diseases. Microglia, ubiquitous within the CNS, prominently feature in the progression of virtually all neurodegenerative conditions, suggesting their potential as a strategic therapeutic target. AAV has emerged as a promising vector for CNS gene therapy delivery. However, the efficiency of AAV-mediated microglial transduction remains suboptimal. While recent advancements have yielded new AAV variants demonstrating enhanced transduction efficacy in rodent models, their translation efficiency to NHP and human subjects is unknown. The imperative for novel AAV variants tailored to target microglia is thus unequivocal, offering a critical avenue for addressing the unmet therapeutic requirements in neurodegenerative disorders.

## Data Availability

No datasets were generated or analysed during the current study.

## Abbreviations

AAV: Adeno-associated virus
AD: Alzheimer’s disease
ALS: Amyotrophic Lateral Sclerosis
AADC: Aromatic L-amino acid decarboxylase
CNS: Central nervous system
CBA: Chicken beta actin
CSF-1R: Colony-stimulating factor 1 receptor
CSF-1: Colony-stimulating factor 1
CMV: Cytomegalovirus
EMA: European Medicines Agency
FDA: Food and Drug Administration
FTD: Frontotemporal dementia
HDLS: Hereditary Diffuse Leukoencephalopathy with Spheroids
HD: Huntington’s disease
INN: International Nonproprietary Name
ICV: Intracerebroventricular
IV: Intravenous
miRNA: MicroRNA
mHTT: Mutant huntingtin protein
NHPs: Non-human primates
PD: Parkinson’s disease
rAAV2: Recombinant AAV2
shRNA: Short hairpin RNA
TM: Triple-mutant
X-ALD: X-linked adrenoleukodystrophy
